# How Could Peers in Online Health Community Help Improve Health Behavior

**DOI:** 10.3390/ijerph17092995

**Published:** 2020-04-26

**Authors:** Yumei Li, Xiangbin Yan

**Affiliations:** 1 Management Science and Engineering, School of Management, Harbin Institute of Technology, 92 Xidazhi Street, Nangang District, Harbin 150001, China; 2 Management Science and Engineering, Dongling School of Economics and Management, University of Science and Technology Beijing, 30 Xueyuan Road, Beijing 100083, China

**Keywords:** online health community, social relationship, social influence, health behavior, social integration, descriptive norm, social support

## Abstract

Human behavior is the largest source of variance in health-related outcomes, and the increasingly popular online health communities (OHC) can be used to promote healthy behavior and outcomes. We explored how the social influence (social integration, descriptive norms and social support) exerted by online social relationships does affect the health behavior of users. Based on an OHC, we considered the effect of three types of social relationships (friendship, mutual support group and competing group) in the OHC. We found that social integration, descriptive norms and social support (information and emotional support) from the OHC had a positive effect on dietary and exercise behavior. Comparing the effects of different social relationships, we found that the stronger social relationship—friendship—had a stronger effect on health behavior than the mutual support group and competing group. Emotional support had a stronger effect on health behavior than informational support. We also found that the effects of social integration and informational support became stronger as membership duration increased, but the effects of the descriptive norms and emotional support became smaller. This study extended the research on health behavior to the online social environment and explored how the social influence exerted by various social relationships in an OHC affected health behavior. The results could be used for guiding users to make use of online social relationships for changing and maintaining healthy behavior, and helping healthcare websites improve their services.

## 1. Introduction

Although many technical breakthroughs have occurred in healthcare recently, human behavior is still the largest source of variance in health-related outcomes [1]. Key healthy behaviors include quitting smoking, a balanced diet, regular exercise and a low alcohol intake [2], which are the most important for keeping healthy. Unhealthy behaviors cause much of the illness, suffering and early deaths related to chronic diseases and conditions [3], which comprise approximately half of deaths in the United States. At the same time, healthcare costs 17.1% of the GDP in the United States and 5.6% of the GDP in China. As health behavior plays a key role in well-being, morbidity and mortality, as well as healthcare costs [1], improving health behavior is the greatest hope for improving the quality of personal life and reducing the burden of preventable diseases and death around the world [4]. Thanks to efforts in public health education and people’s personal interest in learning about health-related issues, most people are aware of the risks of unhealthy behaviors. However, many people continue to engage in unhealthy behaviors for various reasons. It is even more difficult for individuals living with chronic disease, who must maintain a strict level of healthy behavior throughout their lifetime. 

Changing and adhering to a healthy behavior are primarily personal self-management issues for individuals. Many intervention approaches could be used for individuals to promote their healthy behaviors. The social ecological model [5] could be used to summarize such factors that affect health behavior on four levels: individual level, social environment, physical environment and societal factors. The individual level factors directly affect the health behavior, including the demographic characteristic (like gender, level of education, socioeconomic status, etc.) and the psychological factors (like attitudes, motivation, self-efficacy, etc.). The social environment factors comprise social influence brought by various social relationships like organizational characteristics, formal (and informal) rules and regulations in social institutions, formal and informal social networks and social support systems. Physical environment factors include the natural environment (weather or geography) and the man-made environment (availability and access to facilities, community design, public transport). The societal factors are mainly policies on issues such as active transport, education, health or the environment. Since the demographic characteristic, physical environment and the societal factors are difficult for individuals to manage, the psychological factors and social environment factors turn out to be critical for people to adopt and maintain healthy behaviors [6]. Getting support from the social environment to improve ones’ attitude, motivation, self-efficacy and promoting healthy behavior is a great way of changing and adhering to a healthy behavior.

The development of Web 2.0 technology resulted in the dramatic growth of the electronic (online) social environment and blurred the boundaries between the real and the virtual world [7]. Increasingly, social contacts and entertainment happen in the online environment and online social relationships become one of the most important parts of our life. The virtual online social environment has an increasing influence on human beings’ behavior and attracts more and more attention. Following the patient-driven health care model, many Health 2.0 applications combine health information with experience through the use of information and communication technologies, allowing the user to be active and responsible in improving their own health [8]. Health applications are becoming recognized as an effective self-care information sharing and disease self-management tool today [9], which also provides us with a great opportunity to explore the effect of the online social environment on health behavior.

The online health community (OHC) is a popular health application that provides functions of social support, Q&A with physicians, quantified self-tracking and clinical trials access [10]. It provides users with self-entertainment, self-association, self-design, self-discipline and self-healing tools [11]. It is a convenient way to find peers who have experienced similar situations to share information and experiences, or to get personal stories and practical advice [12]. Users can join the OHC to make use of the power of social influence in the OHC to pursue a healthier condition. Communication in the online social environment is characterized by physical separation, anonymity, temporal flexibility and the absence of non-verbal communication [13]. The online social environment allows users to extend their social network and communicate with others at any time, ignoring social class, affluence level, education level and many other features of the offline social environment. OHCs also lower the cost (both in money and time) of forming relationships. To help users improve their health, the OHC provides users with various social relationship functions, such as developing friendships or joining groups to support each other or compete with each other. In the offline environment, it is nearly impossible to find so many peers with whom to form relationships and engage in healthy behaviors. 

Many studies have explored how the social influence exerted by social relationships affected health behavior from a number of perspectives. However, most existing research studies are mainly based on the offline environment, which focused on strong social ties, like family members and friends who meet face to face [14,15,16,17,18,19,20]. Researchers also reported that online social relationship bring significant improvements in some aspects of health behavior change [21]. Through encouragement, accessing answers to specific health-related questions and sharing success stories, online social networks motivate individuals to achieve similar goals [22]. Exploring college-aged women’s Facebook use and eating disorders, Walker et al. found that Facebook intensity, online physical appearance comparison and online “fat talk” is positively associated with eating behaviors [23]. Mhasawade et al. studied the role of the built and online social environments in the expression of dining on Instagram [24]. Merchant et al. delivered weight loss intervention content through Facebook to college students with the help of a health coach, but whether the health behavior changed is unknown [25]. Wang et al. examined physical activity intervention programs via Facebook and found that the social network sites yielded some positive psychological effects but the maximization of benefits needed to be studied [26]. Studies on health behavior have mainly focused on the common social network sites like Facebook and Twitter, but online health communities, which focus on health, have received little attention. Furthermore, few works have explored the effects of the mechanisms promoting healthy behavior in online health communities.

In this paper, we attempted to investigate how the social influence of online social relationships affected health behavior. We focused on three sub-questions: (1). How does the scale of the social relationship affect health behavior? Although users of OHCs benefit from social relationships, establishing and managing the relationship take time and effort. Therefore, we may care that there is a proper scale of the social relationship for users. (2). How does observing others’ behavior affect the one’s health behavior? Observational learning is a form of social learning that occurs since childhood, does it still exist in OHCs? Based on the characteristics of OHCs, here we considered the participation in the following social relationships: friendship (two users follow each other), mutual group (a set of users who share similar goals or interests come together to support each other) and competing group (a set of users who share the same goals or interests come together to compete with each other and improve health in a certain time). We explored the scale effect and the observational learning effect from the three types of social relationships. (3) How could the interaction with peers affect one’s health behavior? Users “talk” to each other to get and provide information or emotional support in the OHC. Considering the detailed content of the social support interactions, which was recognized as informational support, emotional support and companionship support [27], we explored the effects of the different types of social support on health behavior. 

The rest of the paper is organized as follows. We introduce the theoretical background, the related works and hypotheses in Section 2. We introduce our study setting and describe our data in Section 3. Section 4 presents the research model. Section 5 reports the results and our analysis. Finally, in Section 6, we conclude our work with a discussion of study implications, limitations and future research directions. 

## 2. Theoretical Foundations

### 2.1. Theoretical Background

Observational learning. The social learning theory encompasses a mechanism through which individuals learn from each other with direct communications and a mechanism of observational learning where the behavior of individuals is influenced by their observation of other people’s choice. Observational learning does not need reinforcement to occur, but instead, requires a social model such as a parent, sibling, friend, or teacher. The OHCs provide a large number of peers for individuals who could become the model.

Self-determination theory (SDT). This is a macro theory of human motivation and personality that focuses on the social-contextual conditions that facilitate versus forestall the natural processes of self-motivation. The SDT proposes that competence, autonomy and psychological relatedness are the basic psychological needs of humans [28]. It specifies that psychological needs are essential needs that individuals must satisfy to thrive, just as people cannot thrive without water and food [29]. Satisfying basic psychological needs could also motivate people to initiate healthy behaviors [29]. Online communication with peers provides an opportunity to satisfy individual psychological needs, which helps to improve healthy behavior. 

Social support theory. Social support is defined as an exchange of resources between at least two individuals and it is perceived by the provider or the recipient to be intended to enhance the wellbeing of the recipient [30]. Referring to the web of social ties that surround individuals, one important function of a social network is providing social support [4]. The connectedness in the network indicates a person’s social embeddedness and how they derive support from the environment [31]. Bambina points out that social support in the OHC includes informational support, emotional support and companionship [27].

### 2.2. Hypotheses Development

The OHC provides a platform for users to self-monitor and interact with peers. The self-monitor provides users with an opportunity to record all the users’ health behavior and the interaction allows users to observe each other’s behavior and support each other. We focused on the effects of the observational learning from others’ behavior and social support from the interaction on health behavior.

Social integration is the involvement level in social relationships and its measures primarily focus on the social networks of individuals, including the network structure (e.g., size, range, density) and the characteristics of ties (e.g., contact frequency) [32]. Low levels of social integration (that is, having no strong social ties) are most deleterious, with higher levels being less advantageous once a threshold level has been reached. Having at least one strong intimate relationship is an important predictor of good health [33]. Although little social integration is not good for health behavior, there are also severe limits on the quantity of social ties [34]. Robin Dunbar proposed a theoretical cognitive limit to the number of people with whom one can maintain social relationships [35]. As friends and other social ties increase, less attention may be paid to health behavior.

**H1:**  *Social integration has a curvilinear (inverted-U) relationship with health behavior.*

Introduced in theory of planned behavior, descriptive norms are a type of social norm that describe what others actually do [36] and they have a significant effect on intentions, which summarize a person’s motivation to act in a particular way and represent how much time and effort they would devote to performing a behavior [37]. People will follow others’ behavior as described by the herd effect [38]. Furthermore, people will feel guilty about not performing a positive behavior in accordance with their surroundings. Social learning theory puts forward that people learn from others through observing their behavior in the social context. Such learned information allows people to adjust their own behaviors appropriately. As one observes others’ behaviors, the user could increase their self-efficacy (belief in one’s ability to succeed in specific situations [39]) and be encouraged. In OHCs, users participate in healthy behavior together and share their progress. They can get descriptive norms from different social ties.

**H2:**  *Descriptive norms in OHCs are positively related to health behavior.*

According to the SDT, people feel that support and intrinsic motivation are what drives them to perform a behavior for the sake of enjoyment [28]. Berkman et al. found that social support could enhance self-efficacy [40]. Cobb found that sustained social support and social influence were required to promote smoking cessation and smoking abstinence in online social networks [41]. Low support from family and friends are barriers to follow-up care behavior for breast cancer patients [42]. Bambina illustrated the difference between OHC social support and traditional social support, noting that the social support in the OHC includes informational support, emotional support and companionship [27]. 

Informational support includes advice, referrals, teaching, information broadcasting/seeking and personal experience [27]. Through providing health-relevant advice and recommendations, the OHC gives users more choices to make their own informed decision about how to behave, satisfying the need for autonomy identified by the SDT [28]. The experiential information from other patients provides users with a window on other’s second opinions, information that is “difficult” to ask directly yet could help people to better understand a health condition and determine their own behaviors [31]. Learning that others have overcome similar conditions could help satisfy the basic psychological need of competence to perform behaviors [28]. According to the SDT, knowledge about health risks and benefits is one of the core determinants for changing individual behaviors and habits [43].

**H3a:**  *Informational support from online social ties positively affects health behavior.*

The emotional support provided by the OHC includes understanding/empathy, encouragement, affirmation/validation, sympathy and caring/concern [27]. Such emotional support affects users’ relatedness need and could help them feel competent to perform behaviors [28]. Lack of emotional support or isolation from other patients can become a barrier to health behavior adherence [44]. OHCs can provide/be a source of support from a large number of persons without time and space limits.

**H3b:**  *Emotional support from online social ties positively affects health behavior.*

Companionship in OHCs includes chatting, humor/teasing and groupies [27], which can satisfy the relatedness need by making individuals feel that there are others who enjoy their presence and that they are a valuable part of something bigger than themselves [45]. Such support can be found in the discussion forum of the OHC and make individuals feel they are not isolated from the world.

The human body, physiological and psychological needs, knowledge level and psychological maturity all change over time. People become increasingly rational and analytical as they mature [46]. OHC participants will get more information and experience about health behavior through information provided by the website and sharing experiences. Users will take advantage of the information to make more rational decisions. People will tend to guide themselves based on information instead of following others’ behavior, which is the descriptive norm (which is partially caused by the irrational herd behavior). As they become more analytical, they will value the information more, which may increase the effect of informational support on health behavior and decrease the effect of emotional support. As growth continues, the behaviors will become internalized and less affected by external factors. Therefore, we get the following hypotheses:

**H4a:**  *The effect of descriptive norms on health behavior will decrease with longer membership duration.*

**H4b:**  *The effect of informational support on health behavior will increase with longer membership duration.*

**H4c:**  *The effect of emotional support on health behavior will decrease with longer membership duration.*

**Figure 1:**
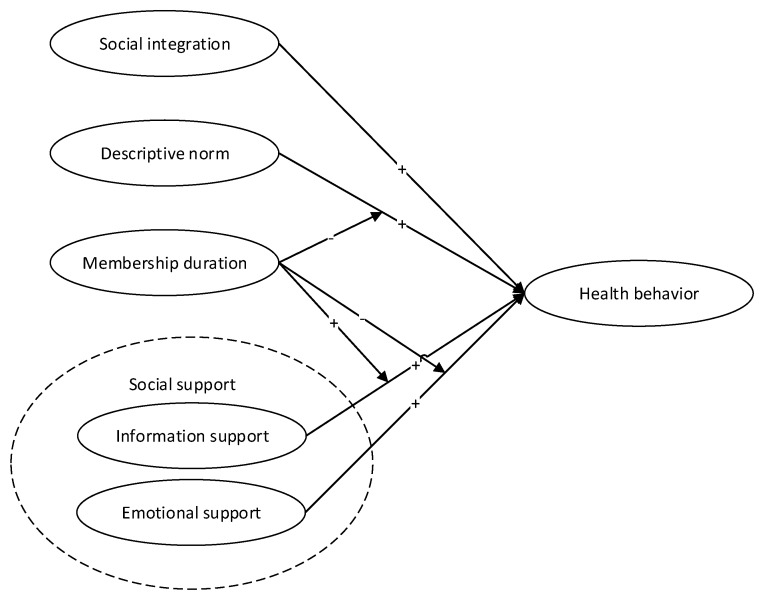
Conceptual framework.

The conceptual framework is showed in Figure 1.

## 3. Materials and Methods

### 3.1. Study Context

Many treatments have been used to address obesity, including surgery [47] (which is expensive, carries some risk and is suitable for only extremely obese individuals), behavior therapy and various dietary approaches [48]. The last two approaches depend on personal behavior changes for successful weight loss. The energy imbalance between the consumed and expended calories is the fundamental cause of obesity [49]; thus dietary and exercise behavior are the most important ways to lose weight.

Our dataset is from a free online weight loss community that provides apps, online tools and community support to help members maintain healthy behavior. Available functions include: (1) finding an available diet, (2) recording their food, exercise and weight diary, (3) establishing online social relationships and (4) exchanging support with others. We crawled members’ information, including personal profile, food diary, exercise diary, weight diary, forum communication and group participation. The detailed data we obtained included: (1) the time the users joined the platform, the starting weight and the target weight; (2) the user’s weight, diet and exercise records, as well as each record time; (3) list of users’ friends, social support groups and competing groups; (4) the group participants in social support groups and competing groups, their diet and exercise records and recording time; (5) the communication data between users and others on the platform (content and time of posts). To clean the data, we deleted some members’ information: (1) members who did not share information, (2) members who did not record/share weight information, (3) members who wanted to lose less than 3kg or whose initial/starting weight was less than 50 kg (to exclude the users who were not really obese or did not want to lose weight). We obtained members’ information from 2011.1.2 to 2011.12.31, spanning 52 weeks. Finally, we obtained 6225 members for our analysis. For the security of the data, we used random numbers to index the users and stored the data on an external disk without connecting with the Internet.

### 3.2. Dependent Variables

Self-monitoring is the centerpiece of behavioral weight loss intervention programs [50], which involves recording the details of health behaviors so that individuals are aware of their current behaviors. Self-monitoring could increase participants’ self-awareness of their targeted behaviors through reminding the participants when the behavior is diminishing and implementing strategies to counteract compliance problems [51]. According to self-regulation theory, self-monitoring precedes a self-evaluation of the progress made towards one’s goal and self-reinforcement for the progress made [52]. Consistently self-monitoring exercise was significantly associated with fewer difficulties with exercise, more exercise and weight loss [51]. Studies that focused on dietary self-monitoring have also found significant associations between self-monitoring and weight loss [50]. Participants with an increased frequency of self-weighing got significantly better weight loss outcomes than those who maintained or decreased their frequency of self-weighing [53].

People who want to lose weight need to focus on healthy diet and exercise behaviors. The main self-monitoring behavior is recording dietary intake and physical activity [54]. We adopted the diet and exercise self-monitoring behaviors as the dependent variables. Everyone had their own definition of healthy behavior, even those with similar conditions, which led to several sets of behaviors. It was difficult to identify whose behavior was healthier. Since the behavior information in OHCs is self-recorded by users, it is frequently incomplete and/or false, leading to incorrect statistical results on calorie consumption. Thus, recorded calorie intake is not a valid measure for health behavior. However, overweight or obese members take care of themselves and try to behave appropriately for their health. The self-monitoring functionality provided by OHCs consists of easy-to-use data entry screens for conditions, symptoms, treatments and other biological information, which will be seen in a graphical display [10]. The more one monitors their health behavior, the healthier their behavior becomes (more exercise and a healthier dietary intake). Therefore, the recorded frequency of diet and exercise in a week is a proper proxy of health behavior. 

We adopted a user’s recorded days in a week (recorded frequency) as the measurement of health behavior participation. If a user recorded their health behavior one day of the week, the user’s recorded frequency in the week will be one. If a user recorded their health behavior all days of the week, the recorded frequency will be seven. If the user did not record health behavior in a week, then the recorded frequency will be zero. Users recorded their diet behavior more frequently than their exercise behavior, but the distribution of the frequency was similar between the diet and exercise behavior. Table 1 presents the statistical information of the users’ recorded frequency in the 52 weeks. Zero has the highest number, and seven is the second highest.

### 3.3. Independent Variables

As discussed in the previous section, we focused on the social relationships in an online social environment, including social integration, social support and descriptive norms, which influenced individuals directly.

Social integration. We used the number of social ties to measure social integration [34]. A social network consists of a series of social ties, which aim to assemble similar members to improve their health together. In OHCs, members connect with each other through friendship and group participation. Two individuals follow each other directly in friendship, whereas a person follows a set of individuals in the group relationship. Therefore, friendship is recognized as a strong tie, and the group relationship is identified as a weak tie in this paper. In OHCs, there are two kinds of groups. (1) The mutual support group, in which people share the same goals or interests to support each other. (2) The competing group, in which people pursue a goal in a given period (it is set as the date the group was created and the period is set for a maximum of three months) at the same time, competing with each other and challenging themselves. Despite the difference in the two kinds of groups, they could both have advantages for users. The mutual support groups provide a warm and accepting interpersonal climate, in which the client is accepted whether or not their goals are achieved [39]. The competing groups show additional rankings for weight loss results, providing a competitive environment. We employed the number of social ties to measure the social integration of social relationships, including the friend number (FNum) and the support group number (SpNum). The establishment of effective social relationships is a dynamic interactive process. It is hard to put a date on when a relationship will start. The interaction is an essential part of establishing a social relationship. We applied the first actual interaction time of two friends as the time of the friendship formation. The interaction included commenting and voting on journals, replying to posts and reviewing recipes. The members’ participation time in the mutual support group was set as the first post or reply to a post in the groups. As some friendships did not have interactions and some members in mutual support groups did not post, we also introduced the order of every user’s friendship formation, the order of the group members’ participation time in the group and the users’ participation time in the OHC to refresh the relationship establishment time as well as the challenge group number (ChalNum). As friendship and membership in the support group will last forever, we also included the number of friendships with a duration of less than three months (FNum3M) and the number of groups a user participated in for three months or less (SpNum3M). We chose three months because the duration of the competing group was a maximum of three months.

Descriptive norm. The descriptive norm was the behavior of others, which was recognized as an important variable that affected behavior. In the OHC, the surroundings included friends and other group members. Therefore, we used the recorded frequency summation of a user’s friends (FDietNum, FExerNum), the recorded frequency summation of a user’s mutual support group members (SpDietNum, SpExerNum) and the recorded frequency summation of a user’s competing group members (ChalDietNum, ChalExerNum) to represent the descriptive norms.

Social support. In the OHC, users made friends and participated in groups to communicate with others and pursued the goal of becoming healthier. In this paper, we identified social support as informational support (InfoSp), emotional support (EmotSp) and companionship (CompSp), and coded the messages according to Bambina’s research [43]. Members in the OHC communicated with each other in the forum and exchanged social support with others through posting messages. We recognized the detailed support content (informational support, emotional support or companionship) contained in the messages by making use of the Sentiment Analysis in LingPipe [55]. We computed the quantity of social support members received according to the characteristics of the communication in the online forums (details are in the Appendix A). 

Member variables. The member’s special information significantly affected their behavior. We introduced the following variables: (1) The member’s initial weight (InitWeight), which was the initial condition of individuals. We could infer that the member with a higher initial weight may have had a higher level of motivation to participate in healthy behavior. (2) The member’s expected weight loss ratio (ExpLossRatio), which was the initial goal for healthy behavior. (3) The duration of the member’s participation in the OHC (membership). (4) The number of diet changes (DietChangeTs), which reflected the member’s weight loss activity level.

The variable descriptions and data statistics are listed in Table 2.

### 3.4. Research Method

Self-monitoring behaviors, the dependent variables, were the recorded frequency of a member in a week, which was an ordinal variable ranging from zero to seven. More records represented healthier behavior. We introduced the ordered logistic model [56] to relate the latent health behavior level to the user’s monitor frequency. We expressed this model in terms of a latent linear response, where observed ordinal responses $Record_{i,t}$ (DietNum, ExerNum) were generated from the latent continuous responses. The $Record_{i,t}^{*}$ was the latent monitor level for user i at time t for the observed recorded frequency $Record_{i,t}$. We modeled the true recorded frequency as Equation (1). T is the social integration variables (Fnum, SpNum and ChalNum). N is the descriptive norm variables (FDietNum, SpDietNum and ChalDietNum correspond to recorded DietNum; FExerNum, SpExerNum and ChalExerNum correspond to recorded ExerNum). S is the social support variables (InfoSp, EmotSp and CompSp) and M is the member specific variables (InitWeight, ExpLossRatio, Membership, DietChangeTs). k is a set of cut points k1–k6. The observed recorded frequency responses were generated by applying thresholds ks as in Equation (2). We modeled that the probability that user i recorded their health behavior frequency smaller than or equal to s at time t is showed in Equation (3):

(1) $$\begin{array}{c} Record_{i,t}^{*:}=\beta_{1:3}S_{i,t-1}+\gamma_{1:3}T_{i,t-1}+\delta_{1:3}N_{i,t-1}\\ +\alpha_{1:5}M_{i,t}+\eta_{1}P_{i,t}+\varepsilon_{i,t} \end{array}$$

(2) $$Record_{i,t}=\left\{ \begin{array}{l} 0\;if\;Record_{i,t}^{*}\leq k_{1}\\ 1\;if\;k_{1}\leq Record_{i,t}^{*}\leq k_{2}\\ \vdots \\ 7\;if\;k_{6}\leq Record_{i,t}^{*} \end{array}\right.$$

(3) $$\begin{array}{ll} P\left(Record_{i,t}\right) & =P\left(Record_{i,t}^{*}\leq K_{s}\right)\\ & =\frac{1+\exp(K_{s}-\beta_{1:3}S_{i,t-1}-\gamma_{1:3}T_{i,t-1}-\delta_{1:3}N_{i,t-1}-\alpha_{1:5}M_{i,t}-\eta_{1}P_{i,t})}{\exp(K_{s}-\beta_{1:3}S_{i,t-1}-\gamma_{1:3}T_{i,t-1}-\delta_{1:3}N_{i,t-1}-\alpha_{1:5}M_{i,t}-\eta_{1}P_{i,t})}\\ & \left(s=1,2,\ldots ,6\right) \end{array}$$

To control the individual factors we could not observe, we introduced the random-effects ordered logistic model to evaluate the effect of social relationships on health behavior [57]. 

## 4. Results

### 4.1. Main Result

Table 3 shows the estimated results. The variance information factor (VIF) was less than 5 in our models, which showed that multicollinearity did not appear to be an issue. The random effects model could control all time-invariant latent variables that may have influenced the dependent variable [58]. The start weight of the users tended to have a positive relationship with health behavior (α_1_ > 0). As the coefficient of ExpLossRatio was negative (α_2_ > 0), users who aimed to lose a higher proportion of weight were concerned with their diet and exercised less. This could be explained by realizing that a big goal may lower the self-efficacy, which will lead the member to be inactive. The OHC participating time of a user had a negative relationship with their health behavior, which was consistent with previous work; individuals who tried more times to lose weight were more likely to fail. The more times a user changed their diet, the more involved the user was in healthy behavior. Changing diet was a signal of individuals’ degree of eagerness to meet their weight loss goals.

**Social Integration.** The effect of social integration on health behavior varied with the type of social ties, but was consistent for both diet and exercise behavior. FNum first had a negative effect on health behavior and the effect became a U-shaped curvilinear as the FNum (γ1 < 0, γ4 > 0) increased. SpNum had a negative relationship with health behavior (γ5 < 0). These were inconsistent with H1. However, ChalNum had a positive effect on health behavior (γ3 > 0, γ6< 0), and the relationship was shown as an inverted U-shaped curvilinear one, which was consistent with H1. The most obvious difference between the challenge relationships, friendships and the mutual support was the duration of the relationship. We tested whether the duration of the relationship caused the different effect of social integration on health behavior in the next part.

**Descriptive norms.** The coefficients of descriptive norms for friendships and competing groups were positive (δ1 > 0, δ3 > 0). The behaviors affected by one’s social ties were significantly positively related with their health behavior; hypothesis H2a was supported. However, the effects of descriptive norms from mutual support groups were very small, even insignificant compared to the effects of friendships and competing groups. We also explored whether the duration of the relationship led to the different effects of descriptive norms from competing groups and mutual support groups.

**Social support.** InfoSp had a significantly positive relationship with health behaviors (β1 >0 for both diet and exercise behaviors). With a higher level of informational support, users performed a higher level of dietary and exercise behaviors. Hypothesis 3a was supported. EmotSp was significantly positively related with the health behaviors (β2 > 0 for both diet and exercise behaviors). Users who received more emotional support performed a higher level of dietary and exercise behaviors. Hypothesis 3b was supported.

### 4.2. Online Social Relationships’ Effect on Health Behavior over Time

We also considered the effect of online social relationships on health behavior over time, based on the duration of OHC participation. Competing groups lasted no more than three months, but mutual support group membership and friendships would last forever, which may have led to the different effects among the social relationships on health behavior. To explore whether different relationship duration affected health behavior differently, we evaluated the effect of social integration and descriptive norms for the first three months after a friendship was formed and mutual support group participation began. Replacing the *FNum*, *SpNum*, *FdietNum* (*FexerNum*) and *SpDietNum* (*SpExerNum*) in Model 1 with *FNum3M*, *SpNum3M*, *FdietNum3M* (*FexerNum3M*) and *SpDietNum3M* (*SpExerNum3M*) respectively, we obtained Model 2. To explore whether the effect of social relationships on health behavior changed while participating in the OHC, based on Model 2, we added the interaction items of membership duration and social relationship variables in Model 3. The estimation results were shown in Table 4.

#### 4.2.1. The Effect of Online Social Relationship Duration

In Model 2, the social integration of friendship in the first three months of the relationship had a significantly positive effect on health behavior ($\gamma_{1}$ > 0, $\gamma_{4}$ < 0), and the scale of the effect changed as the curvilinear relationship (inverted-U). This was consistent with H1. The social integration of a mutual support group in the first three months had a significantly positive effect on health behavior ($\gamma_{2}$ > 0, $\gamma_{5}$ < 0), and the scale of the effect changed with the curvilinear relationship (inverted-U). This was also consistent with H1. 

#### 4.2.2. Comparing the Importance of Online Social Relationships

To contrast the relative importance between social ties, we multiplied the coefficient of social ties by its mean and compared the results. The social integration of friendship had a larger effect on health behavior than the mutual group and the competing group ($\gamma_{1}\times \bar{FNum}$ > $\gamma_{2}\times \bar{SpNum}$, $\gamma_{1}\times \bar{FNum}$ > $\gamma_{3}\times \bar{ChalNum}$). The mutual group and the competing group had nearly the same importance when comparing $\gamma_{2}\times \bar{SpNum}$ and $\gamma_{3}\times \bar{ChalNum}$. Comparing the descriptive norms from different social ties, the results were similar to social integration. Descriptive norms from friendship had the strongest effect on health behavior among the three social ties and the descriptive norms from the two groups caused a similar effect on health behavior. Multiplying the coefficient by its mean, we found that emotional support was more related to health behavior than informational support ($\beta_{1}\times \bar{InfoSp}$ < $\beta_{2}\times \bar{EmotSp}$).

#### 4.2.3. The Effect of OHC Membership Duration

By including the interaction items of membership duration and social relationship variables, we tested how the effect of social relationships on health behavior changed with membership duration in Model 3. Social integration had a greater positive effect on healthy dietary behavior (γ7 > 0, γ8 > 0, γ9 > 0) with longer membership duration. However, this was not true for exercise behavior.

However, the effect of descriptive norms became smaller with longer membership duration (δ_4_ < 0, δ_5_ < 0, δ_6_ < 0 on both dietary behavior and exercise behavior). H4a was supported.

Social support had a different effect on health behavior as membership continued. The effect of informational support on diet and exercise behaviors (β_5_ < 0) became stronger with a longer membership duration. H4c was supported. However, emotional support had a weaker effect as time passed (β_4_ > 0). H4b was supported.

## 5. Discussion

### 5.1. Interpretation of Findings

The social integration of friendship and mutual support group relationship positively affected health behavior in the early stages of the established relationship, but the effect becomes less positive, even negative, based on the whole data as the relationship continues. H1 is conditionally supported. What’s more, the positive effect of the descriptive norms of friendship and mutual support group on health behavior become larger, especially the effect of mutual support groups. This may be because some relationships still showed up in the data but there was less contact or attention. The effect of social integration for competing groups remains the same.

Descriptive norms from social relationship had positive effect on health behavior. Friendships have the strongest effect on health behavior among the three social ties and the descriptive norms from the two groups caused a similar effect on health behavior. This may be because friendship is a strong social tie compared to groups, and strong social ties cause a larger effect on behavior than weak ties [59]. The effect of descriptive norms becomes smaller with longer membership duration. The behavior motivated by descriptive norms is herding behavior, which is irrationally motivated by emotion. As they become more involved in the OHC, users understand more about the OHC environment and become rational, which may lead to the decreasing effect of descriptive norms.

The emotional support and the informational support were positive related with health behavior. Emotional support was more related to health behavior than informational support, which is consistent with Yan’s work [31]. Social support had a different effect on health behavior as membership continued. The effect of informational support on diet and exercise behaviors becomes stronger with longer membership duration. However, emotional support has a weaker effect as time passes: users will become more rational, so they will be less affected by social norms and emotional support and more affected by informational support. In addition, users will learn more from information, which will reduce irrational behavior.

### 5.2. Contribution and Implications

Most of the previous research on health behavior was based on survey data and the social relationship variables were static as the data was collected at one time. We extended the research on health behavior to online social relationships and explored how health behavior was affected by online social relationships with experimental verification using a set of longitudinal data. By seeking to understand the motivation mechanisms that stimulate healthy behavior, our research has the following implications. We found that more social integration, descriptive norms and social support improve individual health behaviors. For health application designers, more informational support and emotional support should be provided to users, with more informational support for older users and more emotional support for newer users. In addition, they should recommend more active friends for the user and provide more opportunities to access others’ behavior information when designing the health application.

### 5.3. Limitations

This paper has limitations that need further work. Firstly, we only identified the content of social support; the support source was not identified. Social support may be identified from different sources, such as friends or group members. Secondly, we only considered social integration from the number of social ties. Other aspects of social integration that could be considered in the future include communication times and content. As for data limitations, we only considered short-term competing relationships. The effect of longer competing relationships should be examined in the future.

## 6. Conclusions

This study revealed that online social relationships play an important role in promoting healthy behavior. Empirical data was collected from an online weight loss community and the random-effects ordered logistic model was employed to test the proposed hypotheses. The results indicate that social integration, descriptive norms and social support from online social relationship have a positive relationship with users’ health behavior. We explored three types of online social relationships: friendship, mutual support group and competing group. Social integration in an online friendship has a stronger effect than other online social relationships and in mutual support groups and competing groups, social integration has similar effects on health behavior. Similarly, descriptive norms in online friendships have a stronger effect than the other two online social relationships and in online mutual support groups and online competing groups, descriptive norms have similar effects on health behavior. The effects of social integration and descriptive norms in online social relationships are stronger at the beginning. We also explored the effect of membership duration and social relationships on health behavior changes. The effect of social integration on health behavior will become larger as membership continues. However, the effect of descriptive norms on diet behavior will become smaller with longer membership. The effect on health behavior will increase for informational support, but decrease for emotional support.

## Figures and Tables

**Table 1:** Health behaviors recorded frequency.

Frequency	Diet Behavior		Exercise Behavior	
	Number	Percentage	Number	Percentage
0	165,534	84.14%	179,097	91.04%
1	7348	3.74%	4562	2.32%
2	3833	1.95%	2322	1.18%
3	2969	1.51%	1762	0.90%
4	2799	1.42%	1640	0.83%
5	2904	1.48%	1418	0.72%
6	2443	1.24%	1138	0.58%
7	8898	4.52%	4789	2.43%
Total	196,728	100%	196,728	100%

**Table 2:** Variables description and data statistics.

Model Variable	Measurement	Description	Mean	Min	Max	Std. Dev.
Behavior	DietNum	Number of diet diaries at week t	0.643	0	7	1.760
	ExerNum	Number of exercise diaries at week t	0.348	0	7	1.321
Social integration	FNum	Number of friends at week t-1	1.690	0	437	6.223
	Fnum3M	Number of friendships established for no more than three months at week t-1	1.586	0	136	5.651
	SpNum	Number of support groups at week t-1	0.827	0	21	1.528
	SpNum3M	Number of support groups, no more than three months at week t-1	0.126	0	17	0.550
	ChalNum	Number of challenges group at week t-1	0.122	0	35	0.522
Social norm	FDietNum	Average diet diary number of friends at week t-1	1.008	0	190	5.372
	FDietNum3M	Average diet diary number of no more than three months friends at week t-1	0.994	0	145	3.409
	FExerNum	Average exercise diary number of no more than three months friends at week t-1	0.563	0	117	3.362
	FExerNum3M	Average exercise diary number of friends at week t-1	0.725	0	92	2.775
	SpDietNum	Average diet diary number of group at week t-1	283.742	0	8429	529.665
	SpDietNum3M	Average diet diary number of group participating for no more than three months at week t-1	44.825	0	5392	205.432
	SpExerNum	Average exercise diary number of support group at week t-1	160.721	0	5197	305.888
	SpExerNum3M	Average exercise diary number of support group participating for no more than three months at week t-1	25.505	0	3443	118.537
	ChalDietNum	Diet diary number of challenge members at week t-1	18.153	0	3392	90.630
	ChalExerNum	Average exercise diary number of challenge members at week t-1	11.430	0	2238	57.849
Social support	CompSp	Companionship support at week t-1	0.002	0	11.54	0.059
	EmotSp	Emotional Support at week t-1	0.009	0	34.847	0.172
	InfoSp	Informational Support at week t-1	0.012	0	50.217	0.297
Member	InitWeight	The last weight input before 2011.1.2	90.578	100	244.9	23.507
	ExpLossRatio	Expectation loss ratio ^a^	19.164	0.088	67	11.287
	Membership	Months since participated OHC until week t	12.545	0.267	62.233	9.769
	DietChangeTs	Number of diet changes until week t	1.159	1	7	0.466

Note. ^a^ Expectation loss ratio is calculated by 100% * (Weight_Start-Weight_Goal)/Weight_Start, the Weight_Start is the users’ weight at the beginning, and the Weight_Goal is the user’s expected weight.

**Table 3:** Estimation results.

Variables	Diet		Exercise	
	Estimate	Standard Error	Estimate	Standard Error
γ_1_ FNum	−0.0207 **	(0.0072)	−0.0248 ***	(0.0076)
γ_2_ SpNum	0.0489	(0.0352)	−0.0231	(0.0404)
γ_3_ ChalNum	0.2490 ***	(0.0258)	0.1517 ***	(0.0305)
γ_4_ Fnum2	0.0001 *	(0.0000)	0.0001 **	(0.0000)
γ_5_ SpNum2	−0.0098 ***	(0.0027)	−0.0063 *	(0.0031)
γ_6_ ChalNum2	−0.0090 ***	(0.0011)	−0.0062 ***	(0.0011)
δ_1_ FDietNum(FExerNum)	0.0520 ***	(0.0029)	0.0571 ***	(0.0042)
δ_2_ SpDietNum(SpExerNum)	0.0000	(0.0001)	0.0003 ***	(0.0001)
δ_3_ ChalDietNum(ChalExerNum)	0.0014 ***	(0.0001)	0.0027 ***	(0.0002)
β_1_ InfoSp	0.2113 ***	(0.0442)	0.1390 ***	(0.0402)
β_2_ EmotSp	0.5968 ***	(0.0589)	0.4627 ***	(0.0557)
β_3_ CompSp	0.2637	(0.1465)	−0.0428	(0.1425)
α_1_ InitWeight	0.0088 ***	(0.0020)	0.0042 ***	(0.0022)
α_2_ ExpLossRatio	−0.0052 ***	(0.0001)	−0.0052 ***	(0.0001)
α_3_ Membership	−0.1564 ***	(0.0027)	−0.1573 ***	(0.0034)
α_4_ DietChangeTs	0.8366 ***	(0.0515)	0.7360 ***	(0.0635)
Number of obs	196,728	196,728
VIF	2.56	2.46
Log likelihood	−104906.87	−65733.06

*Note.* Fnum2 is the square of Fnum, SpNum2 is the square of SpNum, ChalNum2 is the square of ChalNum. *** *p* < 0.001, ** *p* < 0.01, * *p* < 0.05, the standard error is in the parentheses.

**Table 4:** Estimated results with time effect.

Variables	Diet				Exercise			
	Model 2		Model 3		Model 2		Model 3	
	Estimate	Standard Error	Estimate	Standard Error	Estimate	Standard Error	Estimate	Standard Error
γ_1_ FNum3M	0.0694 ***	(0.0161)	0.0547 ***	(0.0169)	0.0461 **	(0.0166)	0.0465 *	(0.0190)
γ_2_ SpNum3M	0.2012 ***	(0.034)	0.1662 ***	(0.0398)	0.1563 ***	(0.0424)	0.0757	(0.0463)
γ_3_ ChalNum	0.2154 ***	(0.0258)	0.1415 ***	(0.0359)	0.1129 ***	(0.0305)	0.0788	(0.0411)
γ_4_ Fnum2	−0.0007 **	(0.0002)	−0.0006 ***	(0.0002)	−0.0006 **	(0.0002)	−0.0005*	(0.0002)
γ_5_ SpNum2	−0.0283 ***	(0.0038)	−0.0251 ***	(0.0037)	−0.0354 ***	(0.0057)	−0.0345 ***	(0.0061)
γ_6_ ChalNum2	−0.0091 ***	(0.0011)	−0.0086 ***	(0.0012)	−0.0059 ***	(0.0011)	−0.0058 ***	(0.0012)
δ_1_ FdietNum3M(FexerNum3M)	0.0719 ***	(0.0145)	0.1503 ***	(0.017)	0.1120 ***	(0.0209)	0.1660 ***	(0.0253)
δ_2_SpDietNum3M(SpExerNum3M)	0.0006 ***	(0.0001)	0.0009 ***	(0.0001)	0.0013 ***	(0.0001)	0.0019 ***	(0.0002)
δ_3_ ChalDietNum (ChalExerNum)	0.0014 ***	(0.0001)	0.0018 ***	(0.0001)	0.0027 ***	(0.0002)	0.0031 ***	(0.0003)
β_1_ InfoSp	0.2013 ***	(0.0431)	0.1287 *	(0.0537)	0.1277 ***	(0.0391)	0.0626	(0.0461)
β_2_ EmotSp	0.6515 ***	(0.0577)	0.6921 ***	(0.0772)	0.5141 ***	(0.0537)	0.7390 ***	(0.0750)
β_3_ CompSp	0.3533 ***	(0.1374)	0.1177	(0.2022)	−0.0147 ***	(0.1405)	−0.2047	(0.1927)
γ_7_ FNum*Membership			0.0010 **	(0.0004)			−0.0001	(0.0006)
γ_8_ SpNum*Membership			0.0067 *	(0.0032)			0.0157 ***	(0.0037)
γ_9_ ChalNum*Membership			0.0103 ***	(0.003)			0.0057	(0.0035)
δ_4_ FDietNum(FExerNum) * Membership			−0.0069 ***	(0.0007)			−0.0048 ***	(0.0011)
δ_5_ SpDietNum(SpExerNum) * Membership			−0.0001 ***	(0)			−0.0001 ***	(0)
δ_6_ ChalDietNum(ChalExerNum) * Membership			−0.0001 ***	(0)			−0.0001 **	(0)
β_4_ InfoSp*Membership			0.0101 *	(0.0048)			0.0098 *	(0.0047)
β_5_ EmotSp*Membership			−0.0082	(0.0068)			−0.0342 ***	(0.0072)
β_6_ CompSp*Membership			0.0275	(0.0176)			0.0304	(0.017)
α_1_ InitWeight	0.0090 ***	(0.0020)	0.0090 ***	(0.0020)	0.0042	(0.0022)	0.0042	(0.0022)
α_2_ ExpLossRatio	−0.0052 ***	(0.0001)	−0.0049 ***	(0.0001)	−0.0053 ***	(0.0001)	−0.0051 ***	(0.0001)
α_3_ Membership	−0.1557 ***	(0.0001)	−0.1481 ***	(0.0027)	−0.1597 ***	(0.0032)	−0.1533 ***	(0.0034)
α_4_ DietChangeTs	0.7942 ***	(0.0510)	0.8147 ***	(0.0511)	0.6758 ***	(0.0630)	0.6932 ***	(0.0633)
Number of obs	196,728		196,728		196,728		196,728	
VIF	2.63		4.13		2.54		4.10	
Log likelihood	−104852.52		−104761.22		−65675.016		−65620.46	

*Note.* Fnum2 is the square of Fnum, SpNum2 is the square of SpNum, ChalNum2 is the square of ChalNum. *** *p* < 0.001, ** *p* < 0.01, * *p* < 0.05, the standard error (S.E) is in the parentheses.

## Appendix A

Every post was analyzed from two dimensions: (1) the social support type of content (e.g., informational support, emotional support and companionship); (2) seeking social support or providing social support. The results are listed in the Table. a1 is the probability of containing informational support in the post. a2 is the probability of containing emotional support in the post. b1 is the probability of seeking social support in the post. b2 is the probability of providing social support in the post. The social support type of a post was assumed to be independent with seeking or providing in the post. a1b1 is the probability of seeking informational support in the post.

	Informational support (a1)	Emotional support (a2)	Companionship (a3)
seeking support (b1)	a1b1	a2b1	
providing support (b2)	a1b2	a2b2	

**InfoSp (Received informational support in the forums for user i at week t) is computed as below:** For (topics in which user i participates in on week t){  //user i joins the discussion on the topic started by others and writes post p. The user gets support from posts before post p   If (post p is the first post for user i on the topic)    For (the first 20 posts on the topic: post q is from post 1 to post 20)       If (post q is the first post on the topic) break;       InfoSp = InfoSp + (the a1b2 of the post q) × (the b1 of post p)   Else    For (the 20 posts before post p: post q is from post p–1 to post p–20)       If (the post q is from user i) break;       InfoSp = InfoSp + (the a1b2 of the post q) × (the b2 of post p)   //user i starts the topic and writes post p. The user gets support from posts after post p   for(the 20 posts after post p: post q is from post p + 1 to post p + 20)     If (the post q is from user i) break;     InfoSp = InfoSp + (the a1b2 of the post q) × (the b1 of post p)

**EmotSp (Received emotional support in the forums for user i at week t) is computed as below:.** For (topics in which user i participates in on week t){  //user i joins the discussion on the topic started by others and writes post p. The user gets support from posts before post p   If (post p is the first post for user i on the topic)    For (the first 20 posts on the topic: post q is from post 1 to post 20)       If (post q is from user i) break;       EmotSp = EmotSp + (the a2b2 of the post q) × (the b1 of post p)   Else    For (the 20 posts before post p: post q is from post p–1 to post p–20)       If (the post q is from user i ) break;       EmotSp = EmotSp + (the a2b2 of the post q) × (the b2 of post p)   //user i starts the topic and writes post p. The user gets support from posts after post p   For (the 20 posts after post p: post q is from post p + 1 to post p + 20)     If  (the post is from user i) break;     EmotSp = EmotSp + (the a2b2 of the post q) × (the b1 of post p)

CompSp is computed as the sum a3 in the forums for user i at week t.
